# Longitudinal trajectories of neurodegenerative biomarkers in the blood of patients with chronic heart failure

**DOI:** 10.1093/eschf/xvag202

**Published:** 2026-07-22

**Authors:** Jan Traub, Anna Frey, György Homola, Caroline Morbach, Stefan Frantz, Mirko Pham, Stefan Störk, Guido Stoll, Markus Otto, Patrick Oeckl

**Affiliations:** Department of Internal Medicine I, University Hospital Würzburg, Würzburg, Germany; Comprehensive Heart Failure Center, University Hospital Würzburg, Würzburg, Germany; Department of Internal Medicine I, University Hospital Würzburg, Würzburg, Germany; Comprehensive Heart Failure Center, University Hospital Würzburg, Würzburg, Germany; Comprehensive Heart Failure Center, University Hospital Würzburg, Würzburg, Germany; Department of Neuroradiology, University Hospital Würzburg, Würzburg, Germany; Department of Internal Medicine I, University Hospital Würzburg, Würzburg, Germany; Comprehensive Heart Failure Center, University Hospital Würzburg, Würzburg, Germany; Department of Internal Medicine I, University Hospital Würzburg, Würzburg, Germany; Comprehensive Heart Failure Center, University Hospital Würzburg, Würzburg, Germany; Comprehensive Heart Failure Center, University Hospital Würzburg, Würzburg, Germany; Department of Neuroradiology, University Hospital Würzburg, Würzburg, Germany; Department of Internal Medicine I, University Hospital Würzburg, Würzburg, Germany; Comprehensive Heart Failure Center, University Hospital Würzburg, Würzburg, Germany; Comprehensive Heart Failure Center, University Hospital Würzburg, Würzburg, Germany; Department of Neurology, University Hospital Würzburg, Würzburg, Germany; Department of Neurology, University Hospital Halle-Wittenberg, Halle (Saale), Germany; Department of Neurology, University Hospital Ulm, Ulm, Germany; German Center for Neurodegenerative Diseases (DZNE e.V.), Ulm, Germany

**Keywords:** Chronic heart failure, Mild cognitive impairment, Neurodegenerative blood biomarkers, Neurofilament light chain, Glial fibrillary acidic protein, β-synuclein

## Abstract

**Introduction:**

Chronic heart failure (HF) is associated with mild cognitive impairment (MCI), vascular dementia, and Alzheimer’s disease. Blood-based neurodegenerative biomarkers may support early risk stratification and outcome prediction in HF.

**Methods:**

In the prospective Cognition.Matters-HF cohort study, serum neurofilament light chain (NfL), glial fibrillary acidic protein, and phosphorylated tau protein-181, as well as plasma amyloid-β peptides (Aβ38, Aβ40, Aβ42) and β-synuclein, were quantified longitudinally over 36 months using ultrasensitive immunoassays and immunoprecipitation mass spectrometry. Cerebral magnetic resonance imaging and standardized domain-specific cognitive testing were performed. Multivariable logistic regression was used to assess associations with future MCI, brain structural changes, and 5-year all-cause mortality.

**Results:**

Among 104 patients with chronic HF (10% female, age 64 ± 10 years), all biomarkers except NfL increased significantly over 3 years (*P* < .05). Median annual percentage changes were 36.6% for phosphorylated tau protein-181, 4.6% for glial fibrillary acidic protein, 2.6% for β-synuclein, 0.6% for Aβ38, 0.5% for Aβ40, and 0.3% for Aβ42. After adjusting for clinical confounders, increases in Aβ38 and Aβ42 independently associated with future MCI, pathological white matter hyperintensity progression (>5% per year), and 5-year mortality (all *P* < .05). Glial fibrillary acidic protein levels and phosphorylated tau protein-181 trajectories were independently associated with pathological brain atrophy (>0.3% per year).

**Conclusion:**

In clinically stable patients with chronic HF, longitudinal increases in circulating amyloid-β peptides associated with cognitive decline, structural brain changes, and mortality. Serial biomarker assessment provided stronger prognostic information than baseline measurements alone. Replication in larger cohorts is required.

## Graphical Abstract

**Graphical Abstract:**
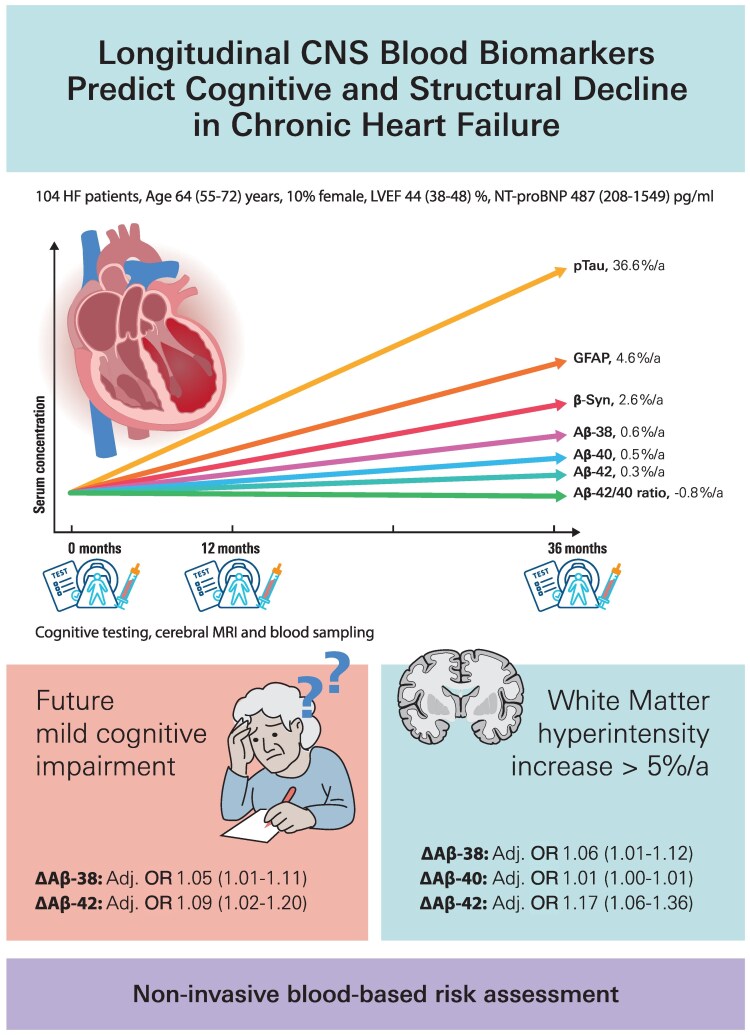

Longitudinal CNS blood biomarker trajectories and their associations with cognitive and structural brain outcomes in 104 patients with chronic heart failure. Biomarkers were measured at baseline, 12 months, and 36 months. Circulating pTau-181, GFAP, β-synuclein, and amyloid-β peptides increased over time, whereas the amyloid-β42/40 ratio decreased. Greater increases in amyloid-β38 and amyloid-β42 were independently associated with future mild cognitive impairment, while increases in amyloid-β38, amyloid-β40, and amyloid-β42 were associated with pathological white matter hyperintensity progression greater than 5% per year.

## Introduction

Chronic heart failure (HF) is a pervasive condition with far-reaching implications, not only for cardiovascular health but also for cognitive function and brain structure.^1^ The underlying mechanisms are complex and multifactorial, involving reduced cerebral perfusion, inflammation, oxidative stress, and neurohumoral changes associated with HF.^2^ Mild cognitive impairment (MCI) is common in HF patients and presents a multifaceted challenge that affects medication adherence, decision-making, caregiver burden, clinical outcomes, and the overall management of HF.^3^ Against this backdrop, central nervous system biomarkers in the peripheral blood may offer a novel and powerful tool for understanding pathophysiological mechanisms, enabling early detection and monitoring MCI progression in HF patients.

In the current study, we present a detailed investigation of longitudinal changes in the concentration of several prominent central nervous system biomarkers in blood samples of a well-characterized prospective HF cohort. NfL (neurofilament light chain) is a marker of neuronal/neuroaxonal damage, Glial fibrillary acidic protein (GFAP) is an indicator of astroglial injury, βSyn (β-synuclein) is associated with synaptic function, pTau (phosphorylated tau) is a marker of tau pathology in neurodegenerative diseases, and Aβ38, Aβ40, and Aβ42 are amyloid-beta peptides linked to amyloid plaque formation in Alzheimer’s disease. Our primary aim was to explore the interrelationships of these blood biomarkers (and their temporal changes) with brain structural changes and cognitive performance over a 3-year period, expanding our previously published baseline results.^4,5^

## Methods

### Study design

The observational, investigator-initiated, prospective, monocentric follow-up study Cognition. Matters-HF was approved by the local ethics committee (study registration number 245/10) and complies with the Declaration of Helsinki. Written informed consent was obtained from all patients. Only patients with confirmed HF according to the guidelines of the European society of Cardiology at study inclusion^6^ were eligible. Newly diagnosed or decompensated HF patients were not suitable. Exclusion criteria incorporated apparent neurological or psychiatric diseases, a history of clinical stroke, carotid artery stenosis over 50%, and devices preventing magnetic resonance imaging (MRI).

### Investigations and follow-up visits

Clinical baseline and 3-year follow-up data have been published before.^7,8^ Patients visited our outpatient clinic at baseline, after 12 and 36 months including blood sampling for routine clinical chemistry and biobank. Physical examination, transthoracic echocardiography, psychological test battery, and brain MRI were performed according to standard operating procedures as published previously.^7,8^ Residents’ registration offices were contacted retrospectively to obtain mortality data after completion of the study. HF phenotypes were classified according to left ventricular ejection fraction (LVEF) as HF with preserved ejection fraction (HFpEF; LVEF ≥50%), HF with mid-range ejection fraction (HFmrEF; LVEF 40–49%), and HF with reduced ejection fraction (HFrEF; LVEF <40%), in accordance with current guideline definitions.

#### Blood sampling and biomarker measurement

We collected blood from all patients by phlebotomy for routine clinical chemistry investigations at the certified facility of the University Hospital Würzburg. Participants were instructed not to fast and were positioned seated for at least 5 min before puncture. Serum and ethylenediaminetetraacetic acid plasma samples were processed within at least 2 h after collection, remained at room temperature for 30 min, and were centrifuged for 10 min at 2000*×g*. Serum and plasma were then aliquoted in specialized fluid tissue tubes (Micronic, Lelystad, Netherlands) and stored in the standardized interdisciplinary biomaterial bank at −80°C until further use.^9^

Serum NfL was measured using the Simple Plex Human NF-L Cartridge (ProteinSimple Ella^TM;^ SPCKB-PS-002448, BioTechne®, Minneapolis, MN, USA). Serum GFAP was quantified with the Simoa GFAP-kit (102 336; Quanterix^TM^, Billerica, MA, USA) and serum pTau using the Simoa pTau-181 advantage V2 kit (104 111; Quanterix^TM^, Billerica, MA, USA) on a Simoa HD-X Analyzer instrument (Quanterix^TM^, Billerica, MA, USA) according to the manufacturer’s instructions. Plasma Aβ38, Aβ40, Aβ42, and βSyn were assessed with immunoprecipitation spectrometry.^10^

Measurements were performed blinded to the patients´ clinical results at the Department for Neurology of the University Hospital Ulm (Ulm, Germany) and the German Center for Neurodegenerative Diseases (Ulm, Germany). Importantly, already published baseline measurements^4,5^ were repeated. Analytical variability was assessed using internal quality control samples. Intra-assay and inter-assay coefficients of variation (CV) were as follows (range across runs): neurofilament light chain (NfL): intra-assay CV 0.6–23.0%; inter-assay CV 8.0–12.9%. Amyloid-β38: intra-assay CV 0.0–5.1%; inter-assay CV 4.4–5.0%. Amyloid-β40: intra-assay CV 0.1–6.7%; inter-assay CV 8.7–9.0%. Amyloid-β42: intra-assay CV 0.3–6.9%; inter-assay CV 12.9–13.1%. Amyloid-β42/40 ratio: intra-assay CV 0.1–9.0%; inter-assay CV 12.0–13.0%. β-synuclein: intra-assay CV 0.6–11.7%; inter-assay CV 4.1–13.1%. Glial fibrillary acidic protein (GFAP): intra-assay CV 1.3–6.9%; inter-assay CV 9.2%. Phosphorylated tau protein-181: intra-assay CV 1.0–14.5%; inter-assay CV 3.8–6.3%. The upper intra-assay CV for NfL reflects a single outlier in a low-concentration quality control sample in one run, while overall inter-assay variability remained within acceptable limits.

#### Cerebral magnetic resonance imaging

Brain MRI was performed with a 3-Tesla scanner (Siemens MAGNETOM Trio, Siemens Healthcare, Erlangen, Germany) using a 12-channel head coil. Magnetization-prepared rapid gradient echo (MPRAGE) data were processed by the FreeSurfer pipeline for segmentation of cerebral structures and comprehensive volumetric analysis.^11^ Measurements of neuro-anatomic structures were determined in mm^3^ or ml, and volumes were normalized to the mean estimated intracranial volume of the cohort. FreeSurfer allowed automatic surface reconstruction and segmentation of brain MRI of arbitrarily many time points. Subcortical brain lesions (white matter hyperintensities, WMH) were determined by segmentation of white matter hypointensities in the T1-weighted MPRAGE data.

#### Cognitive testing

The test battery was performed between 9 and 11 a.m.. Intensity and selectivity of attention were tested by the Test Battery of Attentional Performance, version 2.2. To evaluate visual/verbal memory and working memory, the Visual and Verbal Memory Test (2nd edition), the Digit Span Forward and Block Tapping Span Forward tests of the Wechsler Memory Scale revised were applied. Visual/verbal fluency was analyzed using the Regensburger Word Fluency Test and the HAMASCH-5-Point-Test revised. Premorbid intelligence was used as the control variable to exclude the influence of distinctive intelligence upon the results. Reliability of tests ranged between 0.60 and 0.99. T-standardized output values considered the modifying effect of age, sex, and educational level with a mean of 50 and a standard deviation of 10 points.

### Statistical analysis

Data are displayed as median (interquartile range) or count (percentage). To test for normal distribution, we used the Shapiro–Wilk test. For comparison of independent groups, two-sided *t*-test was applied for normally distributed, Mann–Whitney *U*-test for non-normally distributed data. Chi-squared test was used to find differences between expected and observed frequencies of categorical variables; if the expected value was below five, we used Fischer’s exact test. Correlation analysis was done with Spearman’s correlation. Longitudinal changes over time were evaluated using repeated-measures analysis of variance. This approach was chosen to assess within-subject trends across predefined follow-up time points and is relatively robust to moderate deviations from normality in balanced repeated-measures designs. Nevertheless, this method assumes normally distributed residuals and sphericity, which may not be fully met for all biomarkers with skewed distributions. Univariable and multivariable logistic regression analysis was used to test whether biomarkers were associated with binary outcomes. For selected outcomes, continuous variables were dichotomized using predefined thresholds to allow clinically interpretable logistic regression models. This approach was chosen for exploratory purposes and to limit model complexity given the sample size. All multivariable models simultaneously included age and NT-proBNP as separate covariates to account for age-related increases in NT-proBNP. For the mortality models, covariates were prespecified to reflect HF severity (including NT-proBNP, 6-min walking distance (MWD), renal function, and haemoglobin) while avoiding overfitting given the limited number of events. In sensitivity analyses, inclusion of systolic blood pressure, or LVEF as additional covariates did not materially alter the associations between biomarker trajectories and mortality (data not shown). A two-sided *P* value (*P*) < .05 was considered statistically significant. Due to the explorative character of this study, no correction for multiple testing was done. The few missing values (less than 0.5% missing) were imputed by the median values of the respective variables. Statistical analysis was performed using the statistical software SPSS (version 26) and R (version 4.4.0).

## Results

### Patient characteristics

Of the originally enrolled 148 HF patients, 104 patients had sufficient serum and plasma samples from all three time points (baseline, 12 months, and 36 months) for this *post hoc* biomarker analysis. As shown in *Table 1*, their median age at enrolment was 64 (quartiles 55, 72) years, and 10% were female. Ischaemia was the most common aetiology of HF (66%), with most patients in New York Heart Association (NYHA) functional class II (65%) and a median LVEF of 44 (38, 48)%. Most patients (89%) were treated according to guideline-recommended therapy^6^ at time. The cohort comprised patients across the full spectrum of HF phenotypes, with approximately half classified as HFmrEF, one-third as HFrEF, and a smaller proportion as HFpEF. As published earlier,^8^ during the 3-year observation period, NYHA class II consistently remained the most common, with no changes observed in the 6-MWT distance or LVEF over time.

**Table 1:** Patient characteristics according to the presence of mild cognitive impairment at baseline

Baseline parameter	All patients	None	MCI	*P* value
Number of patients	104 (100)	46 (44)	58 (56)	
Age (years)	64 (55–72)	62 (55–70)	67 (57–73)	.140
Female sex	10 (10)	2 (5)	8 (14)	.179
Body mass index (kg/m^2^)	29 (27–32)	29 (27–32)	29 (26–32)	.140
Systolic blood pressure (mmHg)	138 (125–153)	134 (122–150)	140 (126–156)	.061
Diastolic blood pressure (mmHg)	82 (75–89)	80 (73–87)	82 (75–89)	.154
Heart rate (beats per minute)	62 (58–70)	63 (59–70)	61 (58–69)	.268
Left ventricular ejection fraction (%)	44 (38–48)	44 (38–48)	44 (38–49)	.327
HF with preserved ejection fraction	17 (16)	3 (7)	14 (24)	.**017**
HF with mid-range ejection fraction	51 (49)	25 (54)	26 (45)	.335
HF with reduced ejection fraction	36 (35)	18 (39)	18 (31)	.389
Left ventricular end-diastolic volume (ml)	128 (112–161)	139 (115–166)	123 (109–140)	.134
Left atrial volume index (ml/m^2^)	38 (29–51)	38 (30–53)	37 (27–50)	.474
E/e‘ratio	10 (8–12)	10 (8–14)	10 (7–12)	.133
NT-proBNP (pg/ml)	487 (208–1549)	398 (207–734)	686 (246–1777)	.080
Glomerular filtration rate (ml/min/1.73m^2^)	69 (57–82)	74 (60–86)	65 (56–78)	.084
Six-minute walking distance (m)	420 (360–475)	440 (400–480)	380 (325–440)	.**006**
New York Heart Association class I	25 (24)	17 (37)	8 (14)	.**006**
New York Heart Association class II	68 (65)	28 (61)	40 (69)	.389
New York Heart Association class III	11 (11)	1 (2)	10 (17)	.**012**
New York Heart Association class IV	0 (0)	0 (0)	0 (0)	>.999
Ischemic etiology	69 (66)	27 (59)	42 (72)	.141
Peripheral edema	13 (13)	5 (11)	8 (14)	.486
Fatigue/weakness	38 (38)	14 (30)	24 (41)	.162
Duration of chronic heart failure	3 (1–9)	4 (1–9)	3 (1–9)	.630
Guideline-recommended therapy	92 (89)	44 (96)	48 (83)	.062
Diabetes mellitus type II	30 (29)	12 (26)	18 (31)	.580
Arterial hypertension	89 (86)	38 (83)	51 (88)	.443
Hyperlipidemia	73 (70)	34 (74)	39 (67)	.460
Smoking (ever)	61 (59)	32 (67)	29 (50)	.054
Atrial fibrillation/atrial flutter	19 (18)	8 (17)	11 (19)	.837
Coronary artery disease	73 (70)	29 (63)	44 (76)	.156
History of myocardial infarction	61 (59)	25 (54)	36 (62)	.427
History of coronary artery bypass grafting	25 (24)	9 (20)	16 (28)	.342
Peripheral artery disease	10 (10)	4 (9)	6 (10)	.777
Angiotensin-converting enzyme inhibitor	65 (63)	30 (65)	35 (60)	.610
Angiotensin-1 receptor antagonist	36 (35)	17 (37)	19 (33)	.655
Sacubitril/valsartan	0 (0)	0 (0)	0 (0)	>.999
Beta-blocker	94 (90)	42 (91)	52 (90)	.777
Mineralocorticoid receptor antagonist	40 (39)	18 (39)	22 (38)	.901
Sodium glucose linked transporter 2 inhibitor	0 (0)	0 (0)	0 (0)	>.999
Neurofilament light chain (pg/ml)	23 (15–37)	18 (13–27)	25 (18–38)	.076
Glial fibrillary acidic protein (pg/ml)	182 (116–236)	154 (100–134)	192 (130–236)	.118
Phosphorylated tau-protein 181 (pg/ml)	1.01 (0.36–1.44)	1.04 (0.71–1.25)	0.93 (0.59–1.52)	.258
β-synuclein (pg/ml)	6.4 (4.8–8.2)	6.4 (4.6–7.8)	6.5 (5.3–8.4)	.053
Amyloid-β peptide 38 (pg/ml)	55 (49–62)	54 (47–59)	58 (52–64)	.102
Amyloid-β peptide 40 (pg/ml)	499 (435–572)	473 (402–550)	516 (450–578)	.093
Amyloid-β peptide 42 (pg/ml)	33 (28–37)	32 (28–35)	34 (29–39)	.302
Amyloid-β 42/40 ratio (×10^−3^)	68 (60–72)	68 (63–73)	64 (58–72)	.099

Median (interquartile range) and count (percentage) are displayed, as appropriate. Bold values indicate statistical significance at *P* < 0.05.

### Correlates of absolute biomarker concentrations

**Figure 1:**
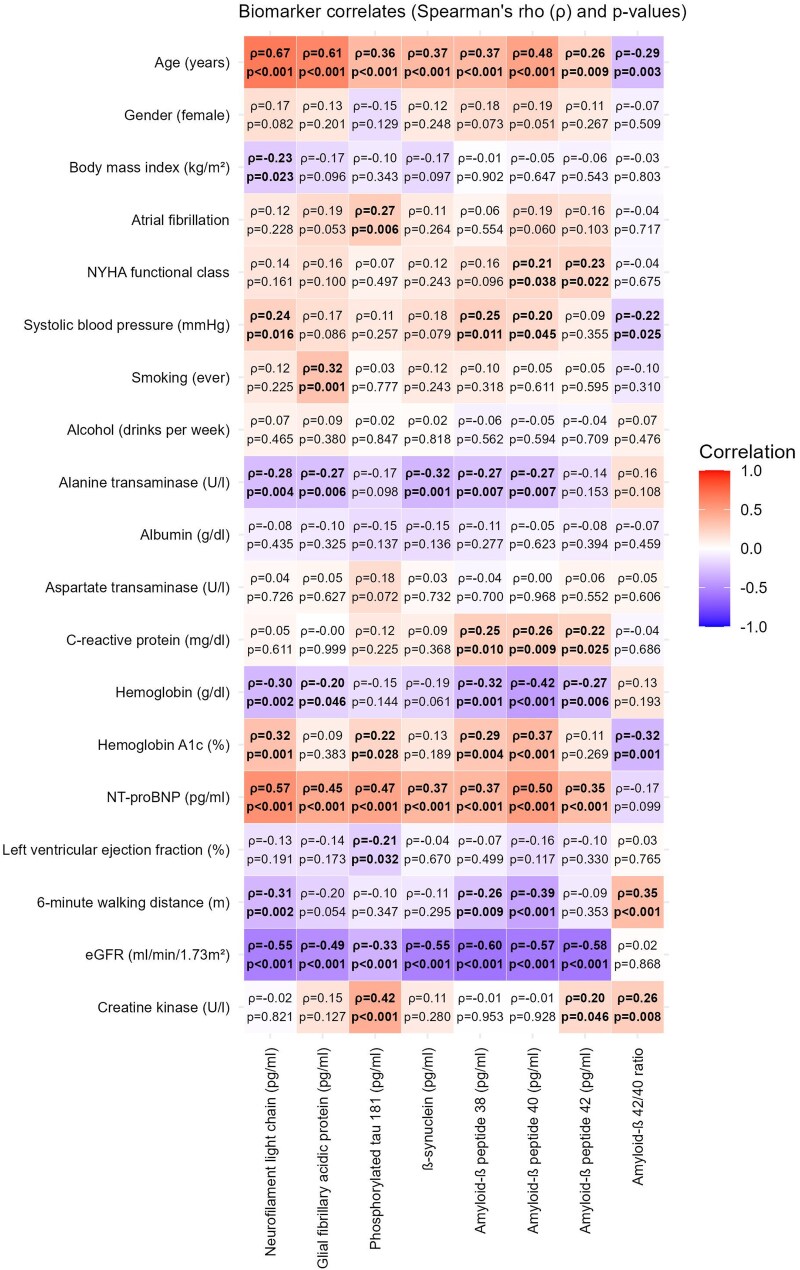
Correlation matrix of absolute biomarker concentrations at baseline with clinical parameters. Spearman’s rho (ρ) correlation was used. (*n* = 104)

First, we tested whether absolute biomarker concentrations at baseline correlated with clinical parameters (*Figure 1*). Indeed, age, smoking status, alanine transaminase, haemoglobin, haemoglobin A1C, NT-proBNP, 6-MWD, estimated glomerular filtration rate (eGFR), and creatine kinase (CK) were significantly related to at least one of the assessed biomarkers. These variables were hence used to control for in multivariable models.

### Longitudinal course of biomarkers, MRI, and cognitive parameters

**Figure 2:**
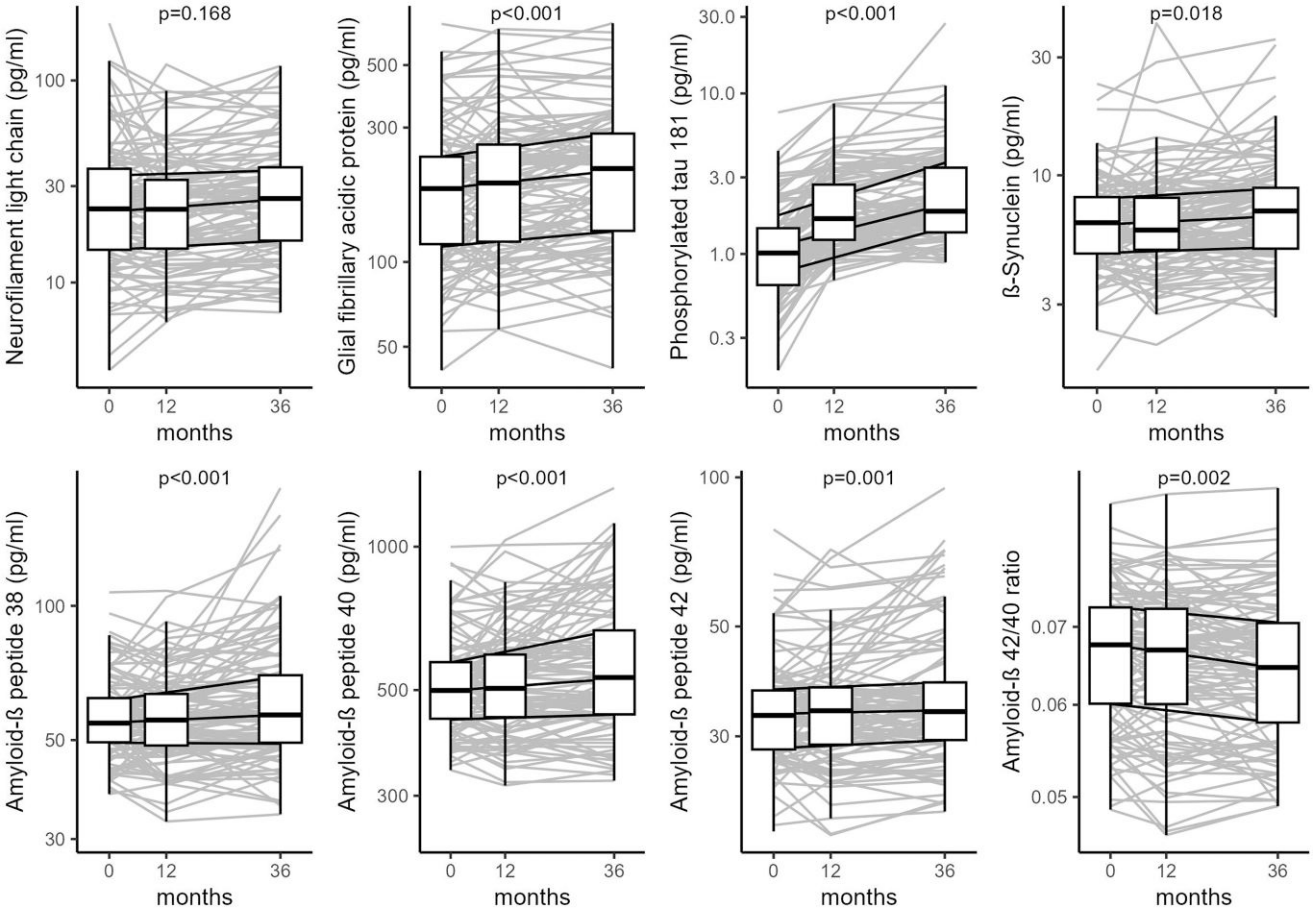
Longitudinal course of blood biomarker concentrations. Grey lines represent trajectories of individual patients. Boxplots display median and interquartile range, whiskers extend to the maximum and minimum data points within 1.5 times the interquartile range. *P* values from repeated measurements ANOVA (*n* = 104)

**Figure 3:**
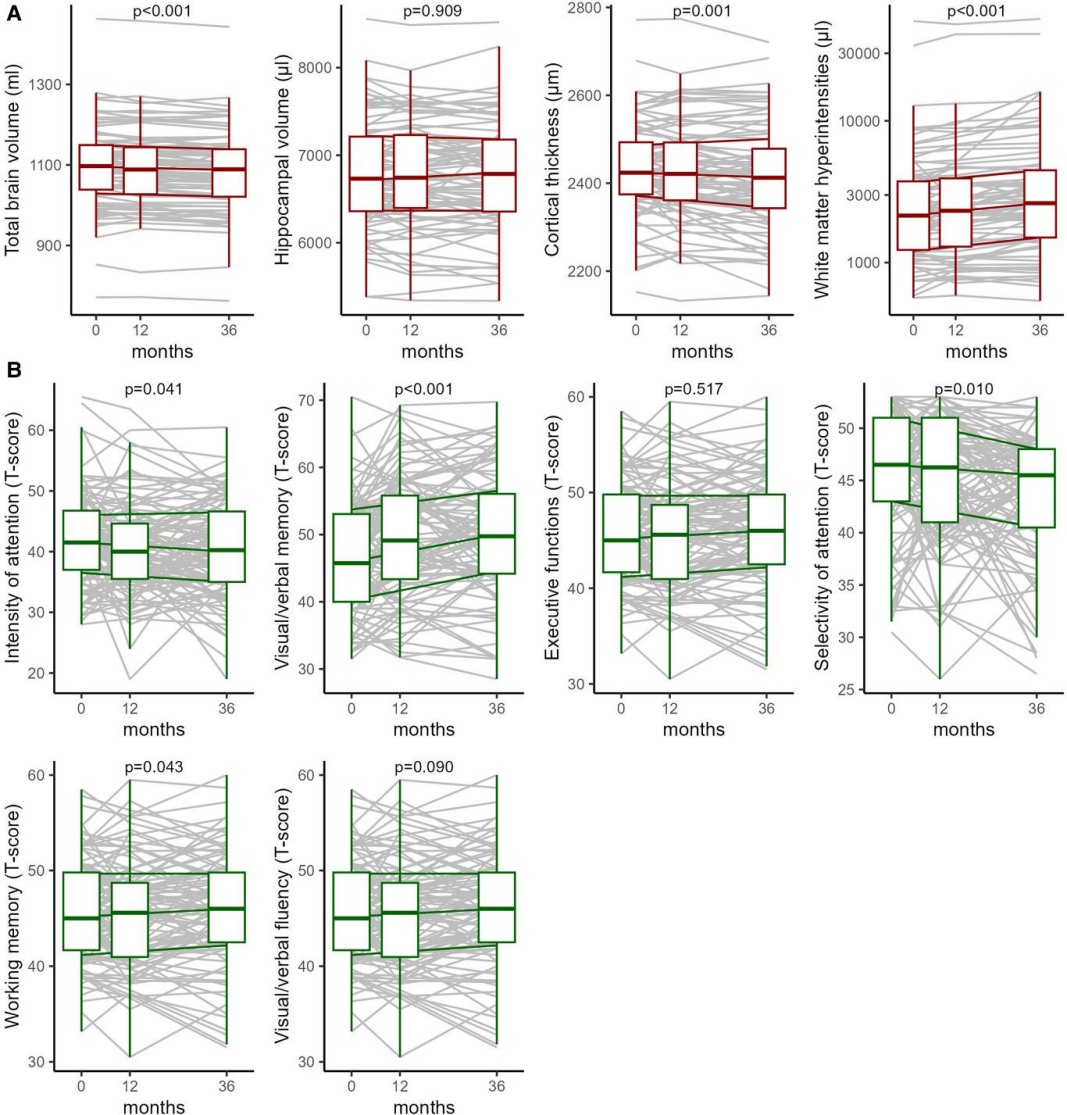
Longitudinal course of brain MRI parameters and cognition. (*A*) Longitudinal changes in total brain volume, hippocampal volume, cortical thickness, and white matter hyperintensity volume. (*B*) Longitudinal changes in cognitive domain T-scores for intensity of attention, visual/verbal memory, executive function, selectivity of attention, working memory, and visual/verbal fluency. Grey lines represent individual patient trajectories. Boxplots display medians and interquartile ranges; whiskers extend to the most extreme data points within 1.5 times the interquartile range. *P* values were derived from repeated-measures ANOVA (*n* = 104)

As shown in *Figure 2*, all biomarkers except NfL increased over the 36-month observation period. The median annual percentage changes were as follows: pTau, 36.6%/a (18.4, 56.8); GFAP, 4.6%/a (−2.0, 15.5); βSyn, 2.6%/a (−3.7, 12.0); Aβ38, 0.6%/a (−2.1, 6.2); Aβ40, 0.5%/a (−0.9, 4.5); and Aβ42, 0.3%/a (−2.1, 4.2). In addition, the Aβ42/40 ratio decreased by −0.8%/a (−2.2, 0.6). While hippocampal volume remained unchanged, total brain volume decreased by −0.3%/a (−0.6, −0.1) and cortical thickness decreased by −0.1%/a (−0.5, 0.2) (*Figure 3A*). Conversely, WMH volume increased by 4.7%/a (1.2, 10.0). During the same period, T-scores for visual/verbal memory and working memory improved, whereas scores for intensity and selectivity of attention worsened (*Figure 3B*).

### Mild cognitive impairment at baseline

**Figure 4:**
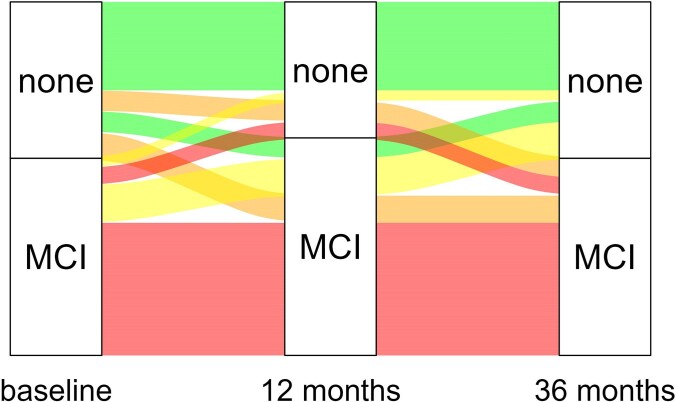
Transition of mild cognitive impairment status over time. This alluvial plot illustrates changes in mild cognitive impairment status from baseline to 12 and 36 months. Each flow represents the longitudinal pathway of patients classified as no mild cognitive impairment throughout follow-up (*n* = 33), transient mild cognitive impairment (*n* = 14), *de novo* mild cognitive impairment (*n* = 13), or persistent mild cognitive impairment (*n* = 44)

At baseline, 56% of this cohort met criteria for test-defined MCI, operationally defined as a T-score <40 in at least one cognitive domain, and not as a clinically adjudicated diagnosis. As shown in *Table 1*, these patients had lower 6-MWD and higher NYHA functional class, while other clinical parameters (including duration of HF prior to enrolment and all assessed biomarkers) did not differ between groups. Forty-four individuals (42% of the cohort) classified as having test-defined MCI at baseline remained with MCI at follow-up. In addition, 13 individuals (13%) who did not initially meet the criteria for test-defined MCI developed the condition over the course of the study (*Figure 4*). This resulted in 57 individuals (55%) with MCI at follow-up (‘future MCI’). Forty-one patients (39%) suffered from MCI at all three assessed time points (‘permanent MCI’).

### Prediction of future and permanent mild cognitive impairment

**Figure 5:**
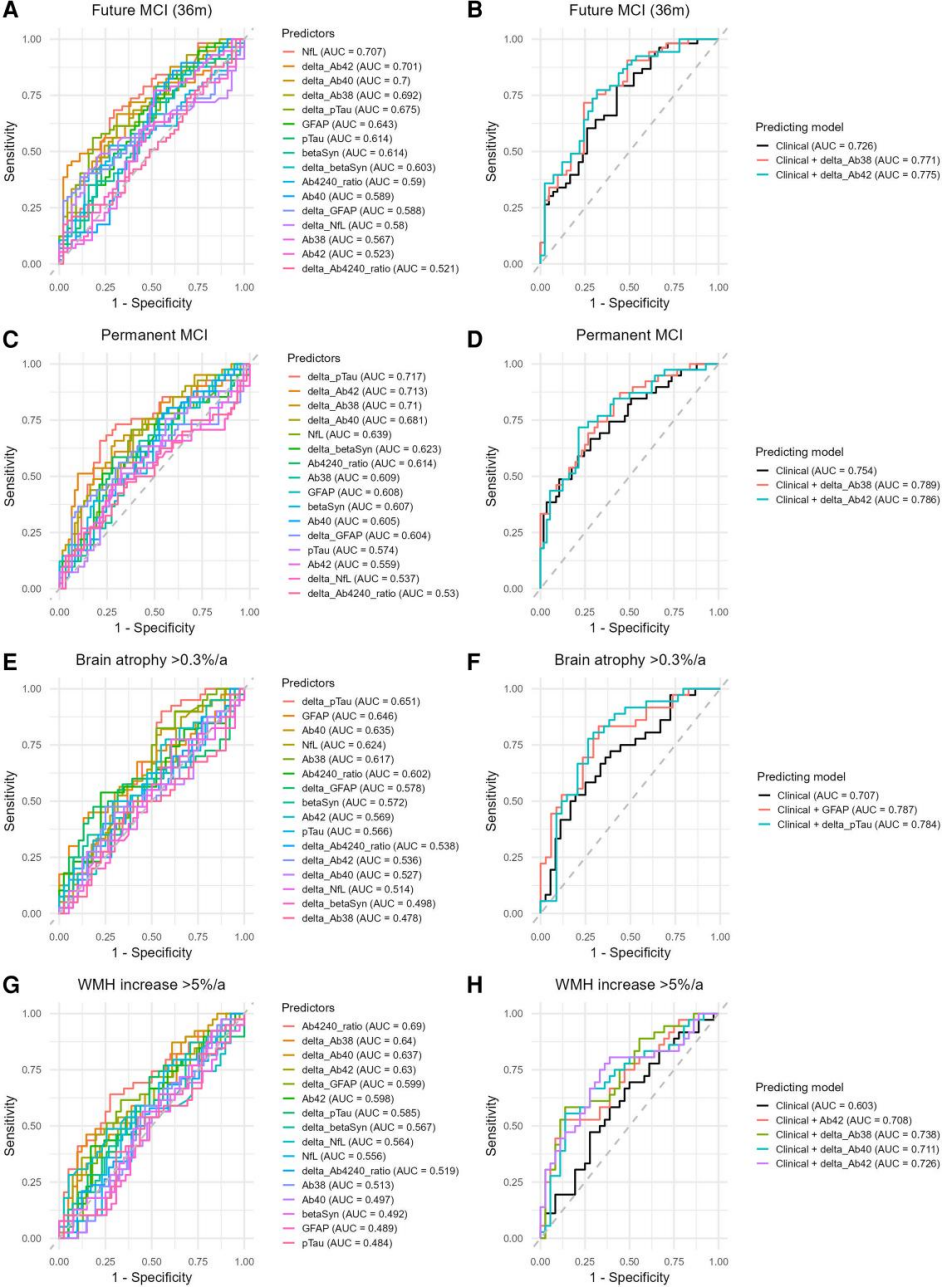
Receiver operating characteristic curves for prediction of cognitive and brain morphological outcomes. (*A*) Discrimination of individual baseline biomarkers and longitudinal biomarker changes for future mild cognitive impairment at 36 months. (*B*) Comparison of the clinical model with models additionally including changes in amyloid-β38 or amyloid-β42 for future mild cognitive impairment. (*C*) Discrimination of individual biomarkers and their longitudinal changes for persistent mild cognitive impairment. (*D*) Comparison of the corresponding clinical and biomarker-extended models for persistent mild cognitive impairment. (*E*) Discrimination of individual biomarkers and longitudinal changes for pathological brain atrophy greater than 0.3% per year. (*F*) Comparison of clinical and biomarker-extended models for pathological brain atrophy. (*G*) Discrimination of individual biomarkers and longitudinal changes for white matter hyperintensity progression greater than 5% per year. (*H*) Comparison of clinical and biomarker-extended models for white matter hyperintensity progression. Areas under the curve are provided in the legends

Univariable logistic regression was used to determine whether biomarkers (or their temporal changes) were associated with future MCI (at 36-month follow-up). As shown in *Table 2*, in univariable regression, this was the case for baseline NfL and GFAP, as well as ΔpTau, ΔAβ38, ΔAβ40, and ΔAβ42. In multivariable models (including the above-identified cofounders age, smoking status, alanine transaminase, haemoglobin, haemoglobin A1C, NT-proBNP, 6-MWD, eGFR, and CK), only ΔAβ38 and ΔAβ42 remained significantly associated with future MCI. *Figure 5A* shows receiver operating characteristic (ROC) curves for individual biomarkers. As shown in *Figure 5B*, the AUC of the ‘clinical model’, which included the above-mentioned cofounders of biomarker levels, improved when ΔAβ38 or ΔAβ42 was added. Similar results were observed for permanent MCI (*Table 3*, *Figure 5C* and *D*). Again, ΔAβ38 and ΔAβ42 were independently associated with this phenotype in multivariable models.

**Table 2:** Logistic regression models for future mild cognitive impairment (at 36-month follow-up)

	Univariable regression			Multivariable regression		
	z value	*P* value	OR (95% CI)	z value	*P* value	Adj. OR (95% CI)
Neurofilament light chain (pg/ml)	**2**.**369**	.**018**	**1.029** (**1.008–1.056)**	1.078	.281	1.012 (0.993–1.042)
Glial fibrillary acidic protein (pg/ml)	**2**.**358**	.**018**	**1.005** (**1.001–1.010)**	1.437	.151	1.004 (0.999–1.011)
Phosphorylated tau-protein 181 (pg/ml)	1.806	.071	1.618 (1.017–2.890)	1.273	.203	1.568 (0.837–3.366)
β-synuclein (pg/ml)	1.905	.057	1.164 (1.011–1.385)	1.108	.268	1.142 (0.921–1.484)
Amyloid-β peptide 38 (pg/ml)	1.019	.308	1.016 (0.986–1.049)	−0.933	.351	0.975 (0.922–1.029)
Amyloid-β peptide 40 (pg/ml)	1.151	.250	1.002 (0.999–1.005)	−0.934	.350	0.997 (0.991–1.003)
Amyloid-β peptide 42 (pg/ml)	0.529	.597	1.012 (0.970–1.059)	−0.952	.341	0.967 (0.900–1.039)
Amyloid-β 42/40 ratio (x10^−3^)	−1.745	.081	0.960 (0.916–1.004)	−0.669	.503	0.981 (0.928–1.037)
Δ Neurofilament light chain (pg/ml)	0.083	.934	1.001 (0.985–1.016)	0.236	.813	1.002 (0.985–1.021)
Δ Glial fibrillary acidic protein (pg/ml)	1.229	.219	1.003 (0.998–1.008)	−0.070	.944	1.000 (0.992–1.007)
Δ Phosphorylated tau-protein 181 (pg/ml)	**2**.**860**	.**004**	**2.108** (**1.312–3.673)**	1.292	.196	1.592 (0.976–3.361)
Δ β-synuclein (pg/ml)	1.956	.051	1.175 (1.024–1.415)	1.446	.148	1.149 (0.983–1.430)
Δ Amyloid-β peptide 38 (pg/ml)	**2**.**890**	.**004**	**1.070** (**1.028–1.127)**	**2**.**002**	.**045**	**1.048** (**1.008–1.107)**
Δ Amyloid-β peptide 40 (pg/ml)	**2**.**633**	.**008**	**1.006** (**1.002–1.010)**	1.846	.065	1.004 (1.000–1.008)
Δ Amyloid-β peptide 42 (pg/ml)	**2**.**876**	.**004**	**1.131** (**1.053–1.245)**	**2**.**085**	.**037**	**1.089** (**1.019–1.201)**
Δ Amyloid-β 42/40 ratio (x10^−3^)	−0.335	.737	0.994 (0.954–1.030)	−0.658	.510	0.986 (0.942–1.028)

Univariable logistic regression models and multivariable regression models (including age, smoking status, alanine transaminase, haemoglobin, haemoglobin A1C, NT-proBNP, 6-MWD, eGFR, and CK) were fitted with z values, *P* values and odds ratios (OR) including 95% confidence intervals (95% CI) being reported (*n* = 104). Bold values indicate statistical significance at *P* < 0.05.

**Table 3:** Logistic regression models for permanent mild cognitive impairment (at all time points)

	Univariable regression			Multivariable regression		
	z value	*P* value	OR (95% CI)	z value	*P* value	Adj. OR (95% CI)
Neurofilament light chain (pg/ml)	**2**.**037**	.**042**	**1.017** (**1.002–1.036)**	0.777	.437	1.007 (0.988–1.028)
Glial fibrillary acidic protein (pg/ml)	**2**.**167**	.**030**	**1.004** (**1.001–1.008)**	1.557	.120	1.005 (0.999–1.011)
Phosphorylated tau-protein 181 (pg/ml)	1.183	.237	1.276 (0.864–1.986)	0.699	.484	1.230 (0.687–2.284)
β-synuclein (pg/ml)	**1**.**983**	.**047**	**1.149** (**1.023–1.340)**	1.128	.259	1.091 (0.964–1.342)
Amyloid-β peptide 38 (pg/ml)	1.278	.201	1.020 (0.999–1.053)	0.669	.503	1.006 (0.991–1.032)
Amyloid-β peptide 40 (pg/ml)	1.035	.301	1.001 (0.999–1.004)	−1.720	.085	0.995 (0.989–1.000)
Amyloid-β peptide 42 (pg/ml)	0.827	.408	1.017 (0.976–1.061)	−1.331	.183	0.954 (0.886–1.020)
Amyloid-β 42/40 ratio (x10^−3^)	0.624	.533	1.003 (0.993–1.021)	0.808	.419	1.008 (0.991–1.044)
Δ Neurofilament light chain (pg/ml)	−0.469	.639	0.996 (0.981–1.012)	−0.117	.907	0.999 (0.982–1.018)
Δ Glial fibrillary acidic protein (pg/ml)	1.597	.110	1.004 (0.999–1.010)	0.358	.720	1.001 (0.994–1.009)
Δ Phosphorylated tau-protein 181 (pg/ml)	0.575	.565	1.047 (0.889–1.291)	−0.360	.719	0.958 (0.651–1.163)
Δ β-synuclein (pg/ml)	1.769	.077	1.113 (1.000–1.275)	1.125	.261	1.091 (0.953–1.303)
Δ Amyloid-β peptide 38 (pg/ml)	**2**.**805**	.**005**	**1.049** (**1.019–1.089)**	**1**.**993**	.**046**	**1.039** (**1.006–1.085)**
Δ Amyloid-β peptide 40 (pg/ml)	**2**.**446**	.**014**	**1.004** (**1.001–1.007)**	1.572	.116	1.003 (1.000–1.006)
Δ Amyloid-β peptide 42 (pg/ml)	**2**.**725**	.**006**	**1.083** (**1.030–1.158)**	**2**.**045**	.**041**	**1.069** (**1.009–1.150)**
Δ Amyloid-β 42/40 ratio (×10^−3^)	0.064	.949	1.001 (0.964–1.041)	0.175	.861	1.004 (0.959–1.052)

Univariable logistic regression models and multivariable regression models (including age, smoking status, alanine transaminase, haemoglobin, haemoglobin A1C, NT-proBNP, 6-MWD, eGFR and CK) were fitted with z values, *P* values, and odds ratios (OR) including 95% confidence intervals (95% CI) being reported (*n* = 104). Bold values indicate statistical significance at *P* < 0.05.

### Prediction of brain MRI changes

Based on the here observed median annual percental changes (*Figure 2*) and elsewhere published changes in normal ageing,^12,13^ dichotomous outcome variables were defined: total brain volume loss >0.3%/a and WMH increase >5%/a. As shown in *Table 4* and *Figure 5E* and *F*, baseline GFAP and ΔpTau predicted pathological brain atrophy independent of clinical variables. Regarding pathological WMH increase, baseline Aβ42, as well as ΔAβ38, ΔAβ40, and ΔAβ42 emerged as independent predictors (*Table 5* and *Figure 5G* and *H*).

**Table 4:** Logistic regression models for pathological brain atrophy (>0.3%/a)

	Univariable regression			Multivariable regression		
	z value	*P* value	OR (95% CI)	z value	*P* value	Adj. OR (95% CI)
Neurofilament light chain (pg/ml)	1.928	.054	1.020 (1.002–1.044)	1.337	.181	1.023 (0.996–1.063)
Glial fibrillary acidic protein (pg/ml)	**2**.**517**	.**012**	**1.007** (**1.002–1.012)**	**2**.**855**	.**004**	**1.014** (**1.006–1.025)**
Phosphorylated tau-protein 181 (pg/ml)	−0.596	.552	0.850 (0.485–1.451)	−1.153	.249	0.604 (0.241–1.386)
β-synuclein (pg/ml)	0.044	.965	1.001 (0.930–1.081)	−0.198	.843	0.988 (0.874–1.109)
Amyloid-β peptide 38 (pg/ml)	−0.382	.702	0.998 (0.985–1.007)	−1.021	.307	0.991 (0.965–1.007)
Amyloid-β peptide 40 (pg/ml)	1.925	.054	1.003 (1.000–1.007)	1.708	.088	1.005 (0.999–1.011)
Amyloid-β peptide 42 (pg/ml)	0.881	.378	1.020 (0.977–1.070)	0.001	.999	1.000 (0.928–1.079)
Amyloid-β 42/40 ratio (x10^−3^)	−1.398	.162	0.962 (0.909–1.000)	−1.743	.081	0.938 (0.869–0.994)
Δ Neurofilament light chain (pg/ml)	−1.047	.295	0.990 (0.968–1.007)	−0.829	.407	0.991 (0.968–1.010)
Δ Glial fibrillary acidic protein (pg/ml)	−0.755	.450	0.998 (0.992–1.003)	−0.482	.629	0.998 (0.990–1.006)
Δ Phosphorylated tau-protein 181 (pg/ml)	**1**.**970**	.**049**	**1.613** (**1.047–2.721)**	**2**.**658**	.**008**	**3.288** (**1.460–8.675)**
Δ β-synuclein (pg/ml)	0.560	.575	1.042 (0.904–1.215)	0.271	.786	1.033 (0.817–1.317)
Δ Amyloid-β peptide 38 (pg/ml)	−0.265	.791	0.997 (0.973–1.020)	−0.068	.946	0.999 (0.963–1.035)
Δ Amyloid-β peptide 40 (pg/ml)	0.625	.532	1.001 (0.998–1.004)	0.197	.844	1.000 (0.996–1.005)
Δ Amyloid-β peptide 42 (pg/ml)	0.263	.792	1.007 (0.956–1.062)	−0.482	.629	0.983 (0.912–1.053)
Δ Amyloid-β 42/40 ratio (x10^−3^)	1.143	.253	1.034 (0.991–1.122)	0.998	.318	1.031 (0.985–1.125)

Univariable logistic regression models and multivariable regression models (including age, smoking status, alanine transaminase, haemoglobin, haemoglobin A1C, NT-proBNP, 6-MWD, eGFR and CK) were fitted with z values, *P* values and odds ratios (OR) including 95% confidence intervals (95% CI) being reported (*n* = 104). Bold values indicate statistical significance at *P* < 0.05.

**Table 5:** Logistic regression models for pathological white matter hyperintensity increase (>5%/a)

	Univariable regression			Multivariable regression		
	z value	*P* value	OR (95% CI)	z value	*P* value	Adj. OR (95% CI)
Neurofilament light chain (pg/ml)	0.346	.729	1.003 (0.988–1.018)	−0.499	.618	0.995 (0.973–1.014)
Glial fibrillary acidic protein (pg/ml)	0.033	.974	1.000 (0.996–1.004)	−0.905	.365	0.997 (0.991–1.003)
Phosphorylated tau-protein 181 (pg/ml)	0.124	.901	1.034 (0.603–1.786)	−0.487	.627	0.837 (0.394–1.720)
β-synuclein (pg/ml)	0.948	.343	1.046 (0.971–1.186)	0.931	.352	1.057 (0.946–1.214)
Amyloid-β peptide 38 (pg/ml)	0.704	.481	1.004 (0.995–1.025)	0.976	.329	1.008 (0.993–1.033)
Amyloid-β peptide 40 (pg/ml)	−0.925	.355	0.999 (0.996–1.002)	−1.919	.055	0.994 (0.988–1.000)
Amyloid-β peptide 42 (pg/ml)	−1.534	.125	0.963 (0.913–1.008)	**−2**.**386**	.**017**	**0.886** (**0.794–0.968)**
Amyloid-β 42/40 ratio (x10^−3^)	0.383	.702	1.002 (0.991–1.019)	0.783	.434	1.007 (0.990–1.032)
Δ Neurofilament light chain (pg/ml)	0.665	.506	1.006 (0.990–1.025)	1.118	.264	1.012 (0.994–1.040)
Δ Glial fibrillary acidic protein (pg/ml)	1.073	.283	1.003 (0.998–1.009)	0.967	.333	1.004 (0.996–1.012)
Δ Phosphorylated tau-protein 181 (pg/ml)	0.115	.909	1.022 (0.695–1.513)	−0.101	.920	0.966 (0.486–1.901)
Δ β-synuclein (pg/ml)	0.427	.670	1.031 (0.894–1.199)	0.577	.564	1.065 (0.861–1.329)
Δ Amyloid-β peptide 38 (pg/ml)	0.978	.328	1.012 (0.990–1.041)	**2**.**151**	.**031**	**1.055** (**1.010–1.115)**
Δ Amyloid-β peptide 40 (pg/ml)	1.574	.115	1.003 (1.000–1.007)	**2**.**046**	.**041**	**1.005** (**1.001–1.011)**
Δ Amyloid-β peptide 42 (pg/ml)	**2**.**089**	.**037**	**1.077** (**1.013–1.167)**	**2**.**494**	.**013**	**1.174** (**1.057–1.360)**
Δ Amyloid-β 42/40 ratio (x10^−3^)	−0.012	.990	1.000 (0.962–1.039)	−0.447	.655	0.991 (0.949–1.034)

Univariable logistic regression models and multivariable regression models (including age, smoking status, alanine transaminase, haemoglobin, haemoglobin A1C, NT-proBNP, 6-MWD, eGFR and CK) were fitted with z values, *P* values, and odds ratios (OR) including 95% confidence intervals (95% CI) being reported (*n* = 104). Bold values indicate statistical significance at *P* < 0.05.

### Prediction of all-cause mortality

**Figure 6:**
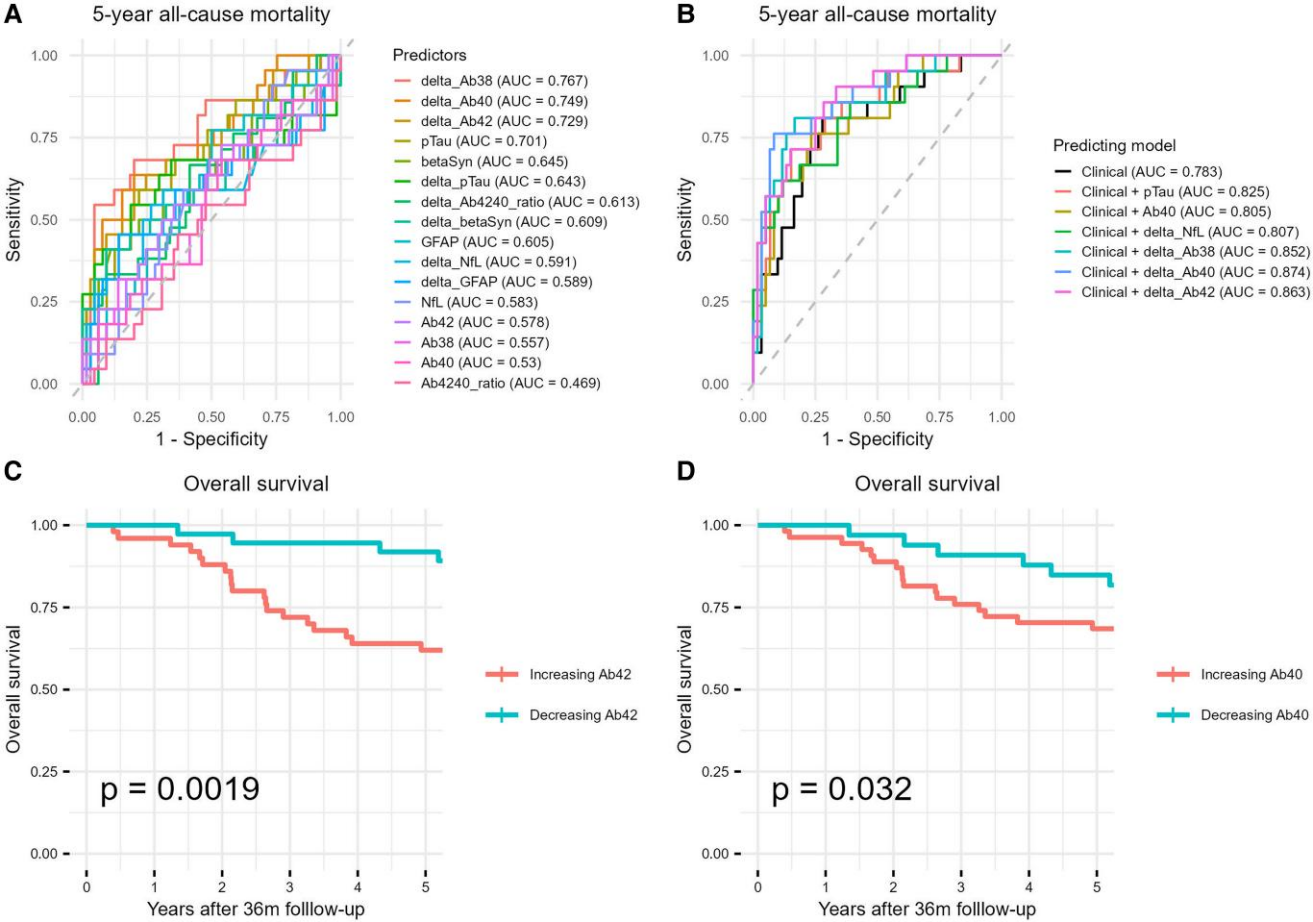
Receiver operating characteristic curves and Kaplan-Meier plots for 5-year all-cause mortality. (*A*) Discrimination of individual baseline CNS biomarkers and their longitudinal changes for 5-year all-cause mortality. (*B*) Comparison of the clinical model with models additionally including selected baseline biomarkers or longitudinal biomarker changes. Areas under the curve are reported in the legends. (*C*) Kaplan-Meier survival curves according to increasing versus decreasing amyloid-β42 concentrations between baseline and 36 months. (*D*) Kaplan-Meier survival curves according to increasing versus decreasing amyloid-β40 concentrations between baseline and 36 months. Survival was assessed during the 5 years following the 36-month visit; *P* values were derived from log-rank tests.

Patients were followed up for at least 5 years after the 36 m follow-up using data from residents’ registration offices. Within this period, 25% (25 of 91 patients) died from any cause. As shown in *Table 6* and *Figure 6A*, baseline pTau and Aβ40, as well as ΔNfL, ΔAβ38, ΔAβ40 and ΔAβ42 predicted 5-year mortality independently and improved clinical models (*Figure 6B*). In line, patients with temporal increasing Aβ40 or Aβ42 concentrations had significantly worse overall survival compared to patients with decreasing concentrations (*Figure 6C* and *D*).

**Table 6:** Logistic regression models for 5-year mortality

	Univariable regression			Multivariable regression		
	z value	*P* value	OR (95% CI)	z value	*P* value	Adj. OR (95% CI)
Neurofilament light chain (pg/ml)	0.560	.576	1.004 (0.988–1.020)	−0.781	.435	0.984 (0.938–1.016)
Glial fibrillary acidic protein (pg/ml)	1.651	.099	1.003 (0.999–1.007)	1.117	.264	1.004 (0.997–1.010)
Phosphorylated tau-protein 181 (pg/ml)	**2**.**654**	.**008**	**2.600** (**1.373–5.561)**	**2**.**575**	.**010**	**3.514** (**1.425–9.992)**
β-synuclein (pg/ml)	1.885	.059	1.144 (1.022–1.340)	1.564	.118	1.151 (0.996–1.435)
Amyloid-β peptide 38 (pg/ml)	0.875	.382	1.016 (0.999–1.056)	1.129	.259	1.012 (0.994–1.056)
Amyloid-β peptide 40 (pg/ml)	0.049	.961	1.000 (0.997–1.004)	**−2**.**046**	.**041**	**0.993** (**0.986–0.999)**
Amyloid-β peptide 42 (pg/ml)	1.491	.136	1.040 (0.987–1.095)	−0.341	.733	0.985 (0.901–1.077)
Amyloid-β 42/40 ratio (x10^−3^)	0.789	.430	1.014 (0.998–1.070)	1.323	.186	1.055 (1.001–1.148)
Δ Neurofilament light chain (pg/ml)	1.873	.061	1.026 (1.002–1.058)	**2**.**372**	.**018**	**1.054** (**1.015–1.108)**
Δ Glial fibrillary acidic protein (pg/ml)	1.032	.302	1.003 (0.997–1.010)	1.020	.308	1.005 (0.996–1.015)
Δ Phosphorylated tau-protein 181 (pg/ml)	1.881	.060	1.529 (1.063–2.460)	1.187	.235	1.530 (0.990–3.202)
Δ β-synuclein (pg/ml)	**2**.**331**	.**020**	**1.174** (**1.042–1.377)**	1.909	.056	1.221 (1.041–1.624)
Δ Amyloid-β peptide 38 (pg/ml)	**2**.**572**	.**010**	**1.033** (**1.011–1.064)**	**2**.**394**	.**017**	**1.054** (**1.017–1.111)**
Δ Amyloid-β peptide 40 (pg/ml)	**3**.**296**	.**001**	**1.007** (**1.003–1.011)**	**2**.**621**	.**009**	**1.010** (**1.004–1.018)**
Δ Amyloid-β peptide 42 (pg/ml)	**3**.**155**	.**002**	**1.115** (**1.052–1.209)**	**2**.**348**	.**019**	**1.140** (**1.049–1.320)**
Δ Amyloid-β 42/40 ratio (x10^−3^)	−0.278	.781	0.993 (0.944–1.058)	−1.502	.133	0.957 (0.899–1.023)

Univariable logistic regression models and multivariable regression models (including age, smoking status, alanine transaminase, haemoglobin, haemoglobin A1C, NT-proBNP, 6-MWD, eGFR and CK) were fitted with z values, *P* values and odds ratios (OR) including 95% confidence intervals (95% CI) being reported (*n* = 91). Bold values indicate statistical significance at *P* < 0.05.

## Discussion

### Key findings

In this longitudinal follow-up study of peripheral CNS biomarkers, including NfL, GFAP, β-synuclein, pTau, Aβ38, Aβ40, and Aβ42 in 104 HF patients, we showed a gradual increase of all biomarkers, except NfL, over the 36-month period with unexpected high dynamics in pTau (+37% per year) and GFAP (+5% per year). Longitudinal increases in Aβ peptides were associated with future MCI, persistent MCI, WMH progression, and all-cause mortality.

### MCI biomarkers in HF

While the detrimental role of MCI in HF patients with regard to quality of life, therapy adherence, and outcome has been established,^14^ only a few studies in this field assessed CNS biomarkers: In 2020, a US American research group linked subclinically lower LVEF to higher pTau-181 and Aβ42 in the cerebrospinal fluid among 152 cognitively normal older adults.^15^ The baseline results of the Cognition.Matters-HF study, connecting serum GFAP with memory deficits^4^ as well as serum NfL and pTau-181 with MCI^5^ were the first reports on peripheral CNS biomarkers in 146 HF patients. These findings were mirrored by two recently published cross-sectional studies: First, a Dutch report with 153 HF patients linked elevated baseline Aβ42/40 ratio and pTau-181 with steeper future cognitive decline.^16^ Second, an Austrian study with 470 HF patients with reduced ejection fraction found independent associations of baseline NfL, total Tau, Aβ40, and Aβ42 with all-cause mortality.^17^

In the present study, we expand these limited findings by longitudinal assessment of seven prominent biomarkers in a well-characterized HF cohort, including cognitive testing and brain MRI. We conclude that changes in Aβ peptide concentrations are associated with MCI, WMH progression, and all-cause mortality.

### Longitudinal vs. cross-sectional biomarker quantification

Our findings suggest that, probably due to their multiple observed clinical correlates (especially age and renal function), serial biomarker measurement is superior to cross-sectional analysis with regard to cognitive, brain morphological and overall outcome. Although recently encouraged by the Alzheimer’s Association,^18^ even in the AD research field, there are few comparable investigations with longitudinal biomarker measurements: Plasma pTau-181 and GFAP increased more rapidly in patients with MCI compared to cognitively normal individuals.^19^ Another trial found that longitudinal trajectories of plasma NfL and pTau-181 among autosomal dominant AD mutation carriers started diverging from trajectories observed for non-carriers at about 16–17 years prior to estimated symptom onset.^20^ Interestingly, plasma pTau-181 started to rise before markers of amyloid pathology became abnormal and associated with prospective AD typical tau aggregation.^21^ In 865 subjects, plasma NfL and pTau-181 concentrations increased along the AD spectrum and showed greater rates of change in AD patients versus controls.^22^ Plasma Aβ42/40 ratio decreased, and plasma p-tau181 and GFAP increased in 27 MCI patients compared with 120 controls over 36 months.^19^

All those studies suggest that these plasma biomarkers may be dynamic in the preclinical phase of AD and highlight the diagnostic and longitudinal monitoring potential for preclinical AD.^23^ Here, we extend those findings to the HF population, which is demonstrably at high risk for vascular dementia and AD.^24^

### Amyloid-β peptides and heart failure

The question arises, why changes in circulating Aβ peptides had highest prognostic ability in our study. Aβ peptides are derived from the cleavage of amyloid precursor protein, and their accumulation is well-known in AD. Interestingly, these peptides, especially Aβ40 and Aβ42, have been identified in the heart tissues of AD patients, suggesting a possible link to cardiac amyloidosis.^25^ Experimental evidence indicates that Aβ deposition in the heart can lead to myocardial dysfunction, fibrosis, and hypertrophy, similar to the effects observed in AD brains.^26^ In this line, the Rotterdam Study reported that higher plasma Aβ40 levels were associated with reduced LVEF, highlighting a potential link between Aβ and cardiac dysfunction.^27^ In our cohort, circulating Aβ peptide concentrations were significantly associated with NT-proBNP levels, further supporting a link between Aβ peptides and HF severity. However, given the observational design of the present study and the absence of disease-specific confirmatory biomarkers for neurodegenerative pathology (e.g. amyloid PET imaging or cerebrospinal fluid measurements), these findings should be interpreted as associative rather than causal. Further research is required to clarify the underlying mechanisms and clinical relevance of these associations.

### White matter hyperintensities, amyloid beta, and mild cognitive impairment

We found a high annual WMH increase of 5% per year, which is in line with the observation that patients with HF often exhibit an increased burden of WMH, being attributed to chronic cerebral hypoperfusion, microvascular dysfunction, and inflammation.^28^ The fact that a change in blood Aβ independently related with higher WMH increase might be explained by the idea that Aβ can deposit in cerebral blood vessels, leading to cerebral amyloid angiopathy,^29^ which exacerbates small vessel disease and contributes to WMH and MCI.

MCI in HF is likely underrecognized in routine clinical practice, as typical symptoms may overlap with HF-related fatigue, depression, or reduced functional capacity and formal cognitive screening is not routinely performed in cardiology clinics. In our cohort, more than half of all patients fulfilled criteria for MCI despite the absence of diagnosed neurological disease, suggesting that the true prevalence of MCI in HF may be substantially underestimated. This highlights the need for increased clinical awareness and systematic cognitive assessment in HF populations.

### Brain atrophy

In line with another publication in 622 in cognitively unimpaired individuals,^30^ Aβ peptides (and NfL) did not predict pathological brain atrophy, which was defined as >0.3% per year, based on MRI studies in normal ageing.^13^ In turn, baseline GFAP and ΔpTau emerged as independent predictors, pointing towards early glial involvement, as well as increasing tau phosphorylation preceding brain atrophy. These findings confirm associations of plasma pTau-181 and GFAP with future brain atrophy in the afore-mentioned report.^30^ The differing associations of biomarkers with structural and cognitive outcomes likely reflect distinct temporal dynamics and underlying biological processes. Baseline GFAP may indicate early astroglial activation preceding measurable neurodegeneration, whereas longitudinal increases in pTau may more closely track ongoing neurodegenerative changes. In contrast, associations of Aβ peptides with WMH progression and cognitive impairment, but not with brain atrophy, may suggest earlier or partly distinct pathways, including vascular-related mechanisms, that may not yet manifest as measurable atrophy in this cohort.

### Strengths and limitations

The study boasts several strengths and limitations that are pivotal for its interpretation. Among its strengths is the longitudinal assessment of biomarkers, cognition, and brain morphology over 3 years, allowing for an in-depth analysis of changes and correlations over time. The study employs a multivariable approach that incorporates clinical confounders, enhancing the robustness and relevance of the findings. Additionally, the use of seven distinct biomarkers measured with cutting-edge technology provides a comprehensive and advanced examination of the subjects’ health status and potential risk factors.

However, the study also has notable limitations. It is conducted at a single centre, which may limit the generalizability of the findings. A lack of positron emission tomography imaging for amyloid deposition and a short observation period of only three years are further limitations of the study. The long storage duration of samples could affect the integrity of the biomarkers. The biomaterials used were inconsistent, with serum NfL, GFAP, pTau, and plasma βSyn and Aβ peptides, which may impede direct comparison of absolute biomarker levels. Furthermore, the low percentage of female participants and the mild HF phenotype present in the cohort might limit the applicability of the results to broader populations. Also, the relatively small sample size necessitates validation in larger cohorts to confirm the study’s findings and ensure their applicability across diverse populations. Because no non-HF control group was included, causal inference regarding HF as an aetiological driver of biomarker changes or cognitive decline cannot be made, and our results should be interpreted as HF-specific risk associations rather than proof of causality.

Another limitation of this study is the use of a purely neuropsychological definition of MCI based on test performance. This operational approach does not incorporate subjective cognitive complaints, functional status, or clinical adjudication and may therefore overestimate the prevalence of MCI compared with consensus criteria, as individuals with low test performance due to normal variability or non-neurodegenerative factors may be classified as impaired. Accordingly, our results should be interpreted as associations with test-defined cognitive impairment rather than clinically diagnosed MCI.

The longitudinal analyses relied on repeated-measures ANOVA, which assumes normality and sphericity and may be less optimal for skewed biomarker distributions. Although this method is relatively robust in balanced designs, future studies may benefit from complementary analyses using linear mixed-effects models or distribution-aware approaches to more flexibly model longitudinal biomarker trajectories.

Given the observational nature of this study and the absence of disease-specific biomarkers such as amyloid positron emission tomography or cerebrospinal fluid measurements, causal or mechanistic conclusions cannot be drawn. The reported associations should therefore be interpreted as descriptive and hypothesis-generating.

Although information on the duration of chronic HF prior to enrolment was available and is reported, this variable was derived from clinical history and may not fully reflect the true onset or cumulative burden of disease. Residual confounding by HF duration therefore cannot be excluded and should be considered when interpreting associations between biomarker trajectories, cognitive impairment, and brain structural changes.

Additionally, we performed dichotomization of continuous outcome variables, including cognitive performance, brain atrophy rates, and white matter hyperintensity progression, using predefined cutoffs. While this strategy facilitates clinical interpretation and comparison with prior literature, it entails a loss of information and may lead to misclassification near threshold values. Consequently, effect estimates should be interpreted with caution, and future studies should consider complementary analyses using continuous outcome measures.

Given the exploratory nature of this study, we did not perform a *post hoc* power calculation. For planning future confirmatory studies, the observed variability of biomarker trajectories and event rates may provide guidance. In this cohort, the standard deviation of ΔAβ38 was 21.9 pg/ml and of ΔAβ42 was 9.8 pg/ml, corresponding to approximate adjusted odds ratios of about 2.8 per 1 SD increase in ΔAβ38 and about 2.3 per 1 SD increase in ΔAβ42 for future test-defined MCI. With a future MCI prevalence of about 55%, confirmatory multivariable models with a similar number of covariates would likely require approximately 180–270 participants to ensure stable estimates. Mortality endpoints, which have lower event rates, would require larger cohorts.

Another limitation is that potential changes in HF therapy during the follow-up period were not systematically recorded and could not be modelled as time-dependent covariates. Although patients were clinically stable and key indicators of HF severity, including LVEF, 6-min walking distance, and NYHA class, remained largely unchanged over time, residual confounding by treatment modifications cannot be excluded and should be considered when interpreting longitudinal biomarker trajectories and outcome associations.

Lastly, no patients in this cohort were treated with angiotensin receptor–neprilysin inhibitors, as recruitment occurred between 2011 and 2014, prior to their introduction into routine clinical practice. As ARNI therapy may influence neurohumoral pathways and disease progression, this limits direct generalizability to contemporary HF populations and should be considered when interpreting biomarker trajectories and outcome associations. Notably, neprilysin is involved in the degradation of amyloid-β peptides, and pharmacological neprilysin inhibition may therefore influence circulating amyloid-β concentrations and their longitudinal trajectories. Consequently, the associations observed between amyloid-β dynamics and cognitive or structural brain outcomes in this pre-ARNI cohort may differ in patients receiving contemporary ARNI-based therapy and should be re-evaluated in future studies.

### Summary and outlook

Taken together, this investigation highlights differential behaviour of several CNS biomarkers in stable HF patients over a 3-year period. Notably, increases in Aβ peptides were associated with future MCI, pathological WMH progression, and 5-year mortality. Future research will be needed to consolidate these findings in larger HF cohorts and explore the potentially pathophysiological role of Aβ (and pTau) in cardiac disease. Importantly, our results identify longitudinal changes in circulating neurodegenerative biomarkers as prognostic associations in chronic HF, rather than evidence of direct mechanistic involvement.

## Declarations

### Data Availability

No data were generated or analyzed for this manuscript.

### Pre-registered Clinical Trial Number

None supplied.
